# A longitudinal study of the post-stroke immune response and cognitive functioning: the StrokeCog study protocol

**DOI:** 10.1186/s12883-020-01897-9

**Published:** 2020-08-26

**Authors:** Lauren L. Drag, Michael Mlynash, Huda Nassar, Elizabeth Osborn, Da E. Kim, Martin S. Angst, Nima Aghaeepour, Marion Buckwalter, Maarten G. Lansberg

**Affiliations:** 1 Department of Neurology and Neurological Sciences, Stanford University Medical Center, 213 Quarry Rd, Palo Alto, CA 94305 USA; 2 Department of Anesthesiology, Perioperative, & Pain Medicine, Stanford University Medical Center, 300 N. Pasteur Dr, Stanford, CA 94305 USA; 3 Department of Neurosurgery, Stanford University Medical Center, 300 N. Pasteur Dr, Stanford, CA 94305 USA

**Keywords:** Stroke, Cognition, Proteomics, Vascular dementia, Neuropsychology, Immune system

## Abstract

**Background:**

Stroke increases the risk of cognitive impairment even several years after the stroke event. The exact mechanisms of post-stroke cognitive decline are unclear, but the immunological response to stroke might play a role. The aims of the StrokeCog study are to examine the associations between immunological responses and long-term post-stroke cognitive trajectories in individuals with ischemic stroke.

**Methods:**

StrokeCog is a single-center, prospective, observational, cohort study. Starting 6–12 months after stroke, comprehensive neuropsychological assessment, plasma and serum, and psychosocial variables will be collected at up to 4 annual visits. Single cell sequencing of peripheral blood monocytes and plasma proteomics will be conducted. The primary outcome will be the change in global and domain-specific neuropsychological performance across annual evaluations. To explain the differences in cognitive change amongst participants, we will examine the relationships between comprehensive immunological measures and these cognitive trajectories. It is anticipated that 210 participants will be enrolled during the first 3 years of this 4-year study. Accounting for attrition, an anticipated final sample size of 158 participants with an average of 3 annual study visits will be available at the completion of the study. Power analyses indicate that this sample size will provide 90% power to detect an average cognitive change of at least 0.23 standard deviations in either direction.

**Discussion:**

StrokeCog will provide novel insight into the relationships between immune events and cognitive change late after stroke.

## Background

Approximately 800,000 individuals in the United States sustain a stroke each year [1]. It is well-known that stroke can be associated with acute cognitive effects, for which some degree of recovery is to be expected. Despite this initial recovery, cognitive impairment occurs at a high frequency, persisting well past the subacute recovery period [2, 3]. Stroke places some individuals at risk of an increased rate of cognitive decline, even several years after stroke, and a history of stroke approximately doubles the long-term risk of incident dementia [2, 4–8].

The pathophysiology underlying post-stroke cognitive decline (and particularly late incident dementia) is not well understood. Some factors have been identified that are associated with increased risk of post-stroke cognitive impairment, such as stroke size and location, increasing age, a lower level of education, a history of pre-stroke cognitive impairment, and cerebral atrophy [9, 10]. In addition, there is a growing evidence that immunological mechanisms may contribute to post-stroke cognitive decline (reviewed in Doyle and Buckwalter, 2020 [11] and Iadecola, Buckwalter, and Anrather, 2020 [12]).

Systemic inflammation is implicated in age-related cognitive decline [13, 14] and also in vascular dementia [15, 16]. Post-stroke dementia is more prevalent with age [5], and is a subset of vascular dementia that may be uniquely related to inflammation. Previous research from our group demonstrated that in animal models, stroke triggers a long-lasting adaptive immune response that is required for post-stroke dementia [17]. Auto-antibodies to myelin basic protein are associated with worsening cognitive trajectory in the first year after stroke [18]. In addition, in both animals and humans, an infection in the days after stroke boosts harmful autoimmunity and worsens overall outcomes, likely by acting as an adjuvant to brain antigens released into the circulation by stroke [19, 20]. Adding to the concept that inflammation in the bloodstream acts as an adjuvant in the presence of brain antigens, we found that immune responses in the acute phase were associated with cognitive trajectories (as measured by a brief cognitive screen) in the year following stroke in a sample of 24 stroke survivors [21]. However, the associations between systemic inflammation and longer-term post-stroke cognitive decline have not been comprehensively studied.

StrokeCog was designed as a single-center comprehensive analysis of post-stroke cognition and systemic inflammation. It is a prospective, observational, cohort study utilizing serial cognitive testing and comprehensive plasma proteomics and single-cell sequencing of peripheral blood mononuclear cells (PBMCs). The primary aims of StrokeCog are to characterize both global and domain-specific cognitive trajectories in the late post-stroke period and to identify the immune determinants of these cognitive changes. The aim of this paper is to describe the design of the StrokeCog study.

## Methods/design

Baseline assessments are conducted between 6 and 12 months following stroke, which is presumably past the early recovery stage and at or near the point of initial stabilization. Participants are then seen for up to 3 additional annual follow-ups over the duration of the 4-year study. The number of annual visits completed by each participant will vary depending on each individual’s time of enrollment across the 4-year study, with a range of 2 to 4 visits and an expected average of 3 visits (SD = 0.82).

**Fig. 1:**
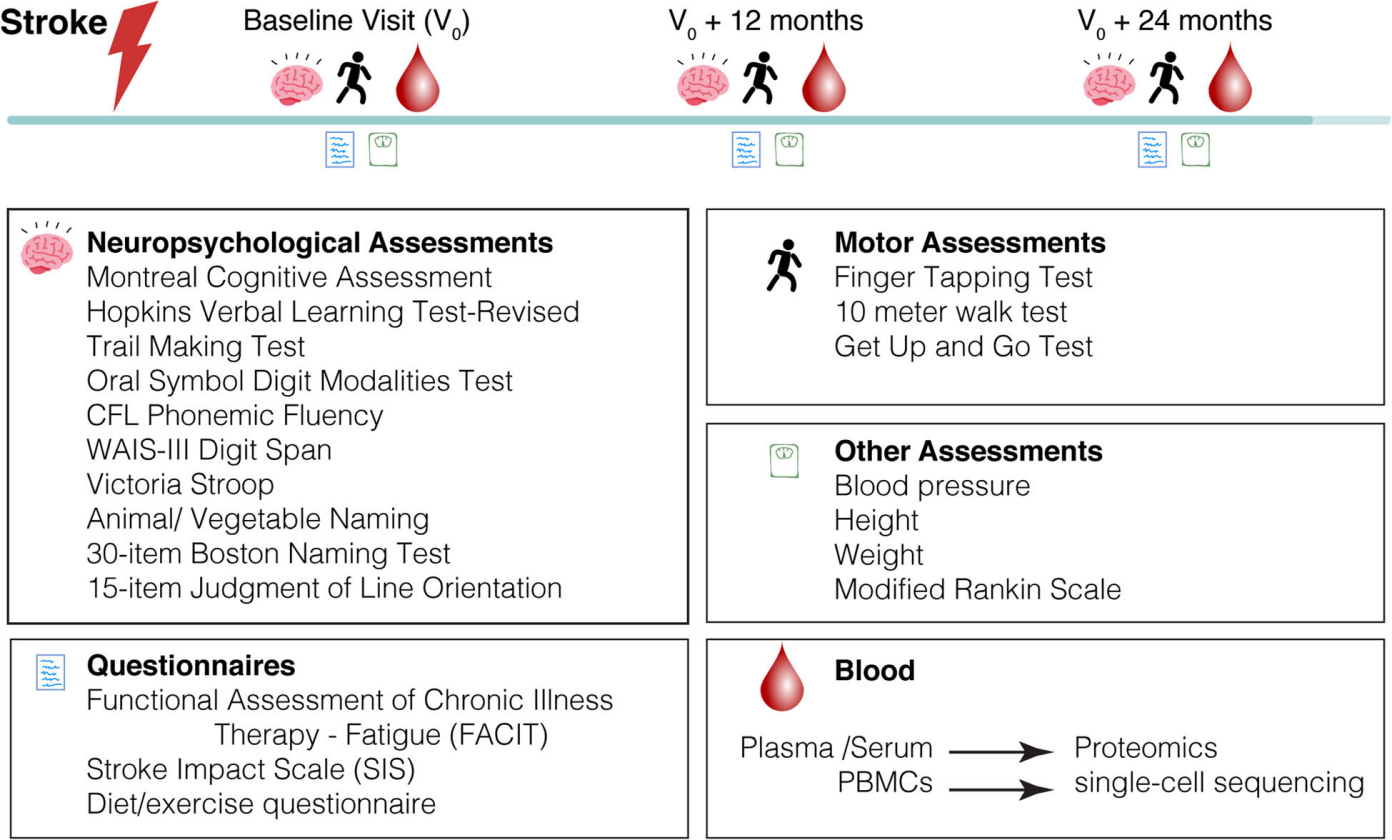
Original schematic of StrokeCog Study design

Participants complete a 60-min neuropsychological assessment, functional questionnaires, motor assessment, and venipuncture at the baseline visit and at all annual visits, scheduled approximately 12 months apart. Assessments conducted at each visit are displayed in Fig. 1.

### Neuropsychological assessment

The neuropsychological test battery was designed based on recommendations for cognitive testing by the National Institute of Neurological Disorders and Stroke and Canadian Stroke Network (NINDS-CSN) [22]. These NINDS-CSN harmonization standards proposed a 5-min, 30-min, and a 60-min neuropsychological battery to examine cognitive domains relevant to vascular cognitive impairment with a particular emphasis on executive functioning and processing speed. For StrokeCog, modifications were made to the proposed 60-min test battery to minimize motor demands for our stroke population. Tests were also selected to optimize overlap with existing test batteries at our site (e.g., Stanford Center for Memory Disorders, Stanford Alzheimer’s Disease Research Center, Pacific Udall Center) to increase potential collaborations and comparisons with other patient populations. The test battery was also designed to utilize alternate versions of tests in successive years when available.

The StrokeCog neuropsychological assessment is a 60-min battery that assesses cognitive domains including processing speed, executive functioning, episodic memory, attention, language, and visuospatial functioning utilizing the following well-validated and standardized neuropsychological tests: Montreal Cognitive Assessment [23] (MoCA; brief mental status screen), Hopkins Verbal Learning Test-Revised [24] (HVLT; learning and delayed recall of a 12-item word list), Trail Making Test [25] (speeded sequencing of numbers and letters), Oral Symbol Digit Modalities Test [26] (speeded matching of numbers and symbols with oral output), Digit Span from the Weschler Adult Intelligence Scale-III [27] (WAIS-III; attention span and working memory), CFL phonemic fluency [28] (speeded word generation in response to phonemic cues), animal/vegetable semantic fluency [28] (speeded word generation in response to semantic cues), 30-item Boston Naming Test [29] (confrontation naming), Victoria Stroop Test [30] (speeded color naming, response inhibition), and 15-item Judgment of Line Orientation [31] (matching of spatial orientations of lines). This test battery yields 15 cognitive variables.

### Questionnaires

The Functional Assessment of Chronic Illness Therapy-Fatigue Scale [32] (FACIT) is a 13-item self-report questionnaire designed to assess fatigue and its impact on functioning. Scores range from 0 to 52 with lower scores indicating more severe fatigue and disability. The Stroke Impact Scale [33] (SIS version 3.0) is a self-report questionnaire covering 8 domains of functioning: strength, cognition, emotional functioning, communication, daily activities, mobility, hand functioning, and participation in meaningful activities. Scores are prorated for any missing values and are summed within each domain with lower scores indicating a higher level of disability.

### Biobanking

Plasma and serum are collected at each visit and aliquoted at − 80 C for proteomics and testing future biomarker candidate genes. PBMCs are isolated and frozen in aliquots for single-cell sequencing and/or DNA.

### Immune measures

We will perform proteomics on plasma using the O-link platform to measure 184 immune and inflammatory proteins. Proteins will be measured using a highly sensitive and specific proximity extension assay. In addition, we will perform single-cell RNA sequencing on PBMCs.

### Motor measures

Gait and fine motor coordination are assessed using the 10-m walk test, the Timed Up and Go Test [34], and the Finger Tapping Test [35].

### Other clinical data

At each annual visit we will record vital signs including height and weight to calculate body mass index, resting heart rate, and blood pressure. A modified Rankin scale [36] is collected to assess functional disability ratings with a score from 0 (indicating no symptoms at all) through 5 (indicating severe disability). Standardized record forms are used to collect a range of demographic and psychosocial data, which include age, ethnicity, primary language spoken, highest level of education, survey of dietary practices, survey of physical activity, alcohol and tobacco use, general health history, and medications.

### Participants

The goal of StrokeCog is to enroll 210 participants with a history of ischemic stroke in the first 3 years of study enrollment. StrokeCog participants are recruited from the Stanford Hospital Inpatient Stroke Unit or the Stanford Stroke Clinic. Inclusion and exclusion criteria were selected to minimize confounding factors (e.g., pre-existing dementia) and other factors that may limit a participant’s ability to complete the study protocol. These criteria are outlined in Table 1. The first participant was enrolled in January of 2019 and recruitment is ongoing.

**Table 1:** Inclusion and exclusion criteria

Inclusion Criteria	Exclusion Criteria
Symptomatic ischemic stroke within the 6–12 months prior to enrollment confirmed by MRI	Score of 2 points or more on the NIH Stroke Scale language component (indicating severe aphasia)
Age 45 years or older	Life expectancy of < 1 year
Known date of stroke (to the month)	Documented diagnosis of dementia predating the stroke
Fluent in English	History of hemorrhagic stroke
Ability to return for annual follow-up visits	Pre-existing neurological, psychiatric, or other conditions (e.g., vision impairment, epilepsy) that would impact assessment of neurologic and/or cognitive outcomes

### Outcomes

For each participant, domain-specific cognitive composite scores and a global cognitive composite score are calculated at each timepoint. The primary outcome is the change in these cognitive composite scores over the study period. Secondary outcomes are the change in depression, fatigue, and functional disability over the study period.

### Sample size estimate

Based on our experience with recruitment of stroke patients from prior studies, we anticipate an enrollment rate of 70 participants per year with approximately 25% of these participants lost to follow-up over the course of the study. This attrition rate is similar to those from other longitudinal studies of post-stroke cognitive assessment [37–39]. Based on these estimates, we anticipate enrollment of 210 participants in the first 3 years of enrollment. Because enrollment will be ongoing throughout the study period, we expect to have 158 participants with at least 2 years of cognitive data available by year 4 of the study.

A power analysis was conducted using prior cognitive data from 66 post-stroke participants who completed at least some of the StrokeCog test battery on 2 occasions, 1 year apart. On average, the annual z-score change of individual neuropsychological measures was 0.01 ± 0.90, while the annual z-score change on the total battery per participant was 0.005 ± 0.341. Based on these calculations and applying a one-sample t-test with H_0_ = 0, a sample size of 158 participants will give us 90% power to detect a z-score change of at least 0.23 on individual measures and a z-score change of at least 0.09 on the total battery per participant.

### Statistical analyses

For the neuropsychological data, raw scores will be transformed to age-corrected (and education-corrected when available) z-scores using manual or published norms, with higher z-scores indicating better performance. To minimize the potential confounds of outlier data on calculations of composite scores, a z-score of − 3 or 3 will be assigned to all z-scores that fall more than 3 standard deviations from the mean. In the event that a participant refuses to complete a test or it is determined based on clinical judgment that the test results are invalid (e.g., due to motor impairment), data will be coded as missing. We anticipate that there will be a subset of individuals who will not be able to complete the neuropsychological assessment due to cognitive impairment (i.e., MoCA score < 10) and will no longer undergo the full neuropsychological battery at their follow-up visits. To avoid biasing the study sample against cognitively impaired individuals, these individuals will be assigned the lowest possible cognitive score (z = − 3) through the remainder of study follow-up.

Using the z-scores from the cognitive variables, a pair-wise undirected Pearson correlation graph (t-distributed stochastic neighbor embedding [TSNE] plot) will be created to visualize the correlation network amongst these cognitive variables. Groupings of variables from the TSNE plot will be used to guide the creation of composite scores for individual cognitive domains. To create composite scores, z-scores for the individual tests within each grouping will be averaged to create an overall composite z-score for that cognitive domain. A global cognitive composite score will also be created by averaging z-scores across domains.

We will apply standard methods for missing data (multiple imputation based on longitudinal models), handling missing data with a variety of missing data mechanisms (missing completely at random [MCAR], missing at random [MAR], missing not at random [MNAR], or combination) but will discard factors and covariates with missingness above 25%.

We will examine predictors of cognitive change for the global and domain-specific composite scores utilizing area under the curve calculations. The primary predictor of interest will be immunological markers (i.e., plasma proteomics and single-cell sequencing data), which will be analyzed using machine learning techniques to provide an unbiased set of predictors of cognitive trajectory. Other predictors that will be entered into the analysis will include demographic factors (e.g., age, gender), stroke factors (e.g., stroke size), and psychosocial and functional factors (e.g., fatigue from the FACIT, functional disability and depression from the SIS).

With machine learning techniques, we will use a multivariate model to find plasma proteins and RNA sequencing data associated with cognitive trajectories. Briefly, for a matrix *X* containing signaling measurements in distinct celltypes (columns) and subjects (rows), and a response vector *Y*, we will apply a supervised algorithm using a linear model to calculate the coefficients ß for each entity in *X* that minimizes overall prediction error L(ß) = |Y-X ß |^2^. However, a linear predictive model with no limitations on ß could choose a complex combination of all measurements that would make interpretation and validation challenging. Model complexity can be reduced minimizing L(ß) = |Y-X ß|^2^ + λ_1_ | ß | _1_ + λ_2_ | *β* |_2,_ where λ_1_ and λ_2_ are selected by cross-validation [40]. This will be done for model interpretation and will produce less expensive models for scaling to larger populations.

## Discussion

StrokeCog is a prospective, observational, cohort study examining the effects of immunological responses on post-stroke cognitive functioning. Cognitive impairment can develop acutely in the immediate aftermath of stroke with some degree of recovery expected over time. However, there is also a risk of a more chronic neurodegeneration as some, but not all, individuals can experience gradual and protracted cognitive decline in the years following stroke at a greater rate than would be expected from aging alone [6, 7]. Consequently, there is an increasing incidence of dementia in the years following stroke [41]. The cost of post-stroke cognitive impairment in terms of loss of quality of life is high as it is predictive of subsequent disability and mortality [10, 42, 43]. This highlights the critical importance of characterizing post-stroke cognitive outcomes, identifying risk factors for cognitive decline, and elucidating the nature and possible mechanisms of post-stroke cognitive impairment [9].

A primary aim of StrokeCog is to provide detailed characterization of long-term cognitive trajectories following stroke over a 4 year period with a 4-year extension that is dependent on additional funding. Some of the larger studies on post-stroke cognition have utilized cognitive screens or clinical diagnosis [8, 41, 44]. However, brief cognitive screens can be limited in both sensitivity and specificity in post-stroke populations [45]. Stroke affects multiple domains of cognitive functioning, although the impact and implications can vary across cognitive domains [6, 38, 46, 47]. As the NINDS-CSN pointed out, executive functioning and processing speed are two cognitive domains that tend to be impacted by stroke [22]. Compared to other cognitive domains, executive functioning and processing speed are associated with a disproportionately higher frequency of impairment, greater rate of decline, and decreased functional outcomes following stroke [6, 48, 49]. Thus, StrokeCog utilizes comprehensive cognitive testing to provide a detailed examination of how cognitive domains are differentially affected by stroke with regard to both severity and trajectory over time. The StrokeCog neuropsychological battery was designed to adhere to NINDS-CSN recommendations as well as to overlap with existing, large-scale datasets with a goal of promoting future collaborations across different studies, sites, and research populations.

A second primary aim of StrokeCog is to examine the relationship between immunological responses and cognitive trajectories. This study is unique in the utilization of novel immunological blood analysis to examine determinants of post-stroke cognitive functioning, which is a promising but under-researched avenue for exploration. The identification of such neurobiological pathways can lead to new insights into potential targets of intervention to attenuate the cognitive decline experienced by many individuals after stroke. Improvement in cognition after stroke has been listed as one of the top ten priorities for post-stroke research [50], yet at the current time, there is limited empirical support for specific interventions to prevent post-stroke cognitive decline, aside from reducing the risk of stroke recurrence. In addition to ameliorating the immediate cognitive effects of stroke, there is a need to prevent future cognitive decline. In fact, the delayed onset of cognitive decline following stroke may potentially represent a therapeutic time window for intervention [9].

Limitations were considered in the design of the study. We are excluding participants with significant aphasia and thus study findings may not be applicable to individuals with large left hemisphere strokes. We also acknowledge that given the requirement for lengthy in-person visits, individuals with higher levels of cognitive and/or motor impairment and disability may be more likely to drop out of the study, biasing the study sample towards a higher functioning population. We plan to mitigate this bias, at least in part, by assigning the lowest possible cognitive scores throughout the remainder of study follow-up for those participants who drop out due to cognitive impairment.

Finally, we acknowledge that due to lack of repeat neuroimaging, we will be unable to identify any individuals who sustain additional subclinical strokes during the study period. We are, however, in the process of obtaining additional grant funding to support follow-up MRI imaging on all subjects.

In sum, the StrokeCog study utilizes serial neuropsychological assessment to characterize long-term cognitive trajectories following stroke. By also examining comprehensive immunological and molecular measures of peripheral blood, this study will provide novel information about relationships between immune makers and domain-specific cognitive changes change late after stroke.

## Data Availability

The datasets used and/or analyzed during the current study are available from the corresponding author on reasonable request.

## Abbreviations

PBMC: Peripheral blood mononuclear cells
NINDS-CSN: National Institute of Neurological Disorders and Stroke and Canadian Stroke Network
MoCA: Montreal Cognitive Assessment
HVLT: Hopkins Verbal Learning Test-Revised
FACIT: Functional Assessment of Chronic Illness Therapy-Fatigue Scale
SIS: Stroke Impact Scale
TSNE: T-distributed stochastic neighbor embedding
MCAR: Missing completely at random
MAR: Missing at random
MNAR: Missing not at random
